# S69. A CASE STUDY OF CLOZAPINE AND COGNITION: FRIEND OR FOE?

**DOI:** 10.1093/schbul/sby018.856

**Published:** 2018-04-01

**Authors:** Gisela Mezquida, George Savulich, Samuel Atkinson, Miguel Bernardo, Emilio Fernandez-Egea

**Affiliations:** 1 Hosp. Clinic Barcel, Institut Clínic Neuro; 2 University of Cambridge; 3 Cambridgeshire and Peterborough NHS Foundation Trust; 4 Hospital Clínic, Universitat De Barcelona/IDIBAPS/CIBERSAM; 5 University of Cambridge, Cambridgeshire and Peterborough NHS Foundation Trust, CIBERSAM

## Abstract

**Background:**

Cognitive dysfunction is as a hallmark feature of schizophrenia. Antipsychotic medication is effective for treating the positive symptoms of psychosis, but their potential for therapeutic effects on cognition continues to divide researchers.

Improvements in clinical symptoms can occur independently of cognitive functioning, whereby typical antipsychotics improve different clinical domains, but have little or no efficacy for improving primary cognitive functions. However, it has been suggested that the second-generation antipsychotic clozapine can improve cognition in schizophrenia. Unusual cases, such as remitted patients who decide to stop taking clozapine, thus represent a unique opportunity to understand the effect of antipsychotic medication on cognition, as described in the case study below.

**Methods:**

A 38-year old man suffered severe psychotic episodes at age 19, leading to a diagnosis of treatment-resistant schizophrenia. Clozapine was initiated at age 21, with excellent treatment response and complete remission of positive, negative and depressive symptoms. For the following 16 years, he had a full-time paid job, lived independently and had an ample social circle. For the last five years, he was stable and compliant taking 175 mg of clozapine, with clozapine levels in sub-therapeutic range (between 0.17 and 0.26 mg/L). He had no metabolic syndrome and no oversedation, but complained of hypersalivation and persistent memory problems. He decided, against advice, that medication was no longer needed and requested support for waning off clozapine.

A five-month program was subsequently implemented, with 25 mg reductions of clozapine every six weeks. Four enhanced assessments were scheduled after two weeks on stable doses of 125, 75, 25 and 0 mg, respectively, which included the Brief Assessment of Cognition in Schizophrenia (BACS) and the Clinical Global Impression-Schizophrenia scale (CGI-SCH).

**Results:**

At each level of clozapine dose, the patient continued to be stable. However, the patient showed impaired cognitive performance at the highest dose of clozapine titration (see T-scores from Table and figure), with improvements then shown at each level of dose reduction (75 and 25 mg). The final assessment was done after 6 weeks of not taking medication. At this time, he was admitted to hospital for psychotic relapse. Unfortunately, the patient refused to re-initiate clozapine and has remained floridly psychotic for 18 months.

Our case suggests that cognitive impairment is dose-dependent with clozapine. As presented, the t-score of the patient at 125 mg was worse than normative data reported for healthy controls (z-score=0.50) and patients with schizophrenia treated with other first-or second generation antipsychotics (z-score=-1.42). However, cognitive performance of the patient was most notably impaired when medication-free.

**Discussion:**

The longterm effects of high-dose antipsychotic medication on cognition in patients with schizophrenia are largely unknown. It is also possible that changes in cognitive states are either domain-specific with clozapine. Nonetheless, daily antipsychotic dose and polypharmacy have been shown to predict poor cognitive functioning.

The present case is also a reminder that the appropriate long-term maintenance dose of clozapine is poorly understood. As this case suggests, the current recommended therapeutic levels of clozapine (0.35 to 0.50 mg/L) might be unnecessarily high for patients in full remission. Controlled studies are therefore needed to evaluate the potential for cognitive improvements in schizophrenia as a function of reduced clozapine dose, to ensure the optimal delivery needed not just for good clinical but also good cognitive outcome.

